# Identification of a crucial *INO2* allele for enhancing ethanol resistance in an industrial fermentation strain of *Saccharomyces cerevisiae*

**DOI:** 10.1128/msystems.00827-25

**Published:** 2025-10-28

**Authors:** Sonia Albillos-Arenal, Javier Alonso del Real, Ana Cristina Adam, María Lairón-Peris, Eladio Barrio, Amparo Querol

**Affiliations:** 1 Yeastomics Laboratory (SBYBI Group), Food Biotechnology Department, IATA-CSIC, València, Spain; 2 Departament de Genètica, Universitat de València 16781, Valencia, Spain; Génomique Métabolique, Genoscope, Institut François Jacob, Evry, France

**Keywords:** *S. cerevisiae*, ethanol stress, transcriptomics, membrane lipids, inositol

## Abstract

Ethanol toxicity is a major challenge for *Saccharomyces cerevisiae* during fermentation, affecting its growth and influencing the process. This study investigated the molecular mechanisms of ethanol tolerance using transcriptomics analysis of three *S*. *cerevisiae* strains chosen due to their differing levels of resistance to ethanol described in a previous work, which linked them to differences in their membrane compositions. Transcriptomic analysis revealed distinct responses in membrane lipid synthesis genes, particularly those involved in ergosterol biosynthesis, in ethanol-tolerant strains carrying a variant of the *INO2* allele. This variant, which includes V263I and H86R amino acid replacements in the Ino2p transcription factor, was exclusive to ethanol-tolerant strains. CRISPR-Cas9-mediated reversion of the variant *INO2* allele to the wild-type sequence in the highly tolerant strain AJ4 resulted in decreased ethanol tolerance. Our findings demonstrate the crucial role of Ino2p in ethanol tolerance through its regulation of lipid synthesis and membrane composition, highlighting the complex interplay of transcription factors in strain-specific ethanol resistance.

**IMPORTANCE:**

This study provides critical insights into the molecular basis of ethanol tolerance in *Saccharomyces cerevisiae*, a key trait for improving industrial fermentation processes. By identifying specific genetic variants in the Ino2p transcription factor and their impact on ethanol resistance, we reveal potential targets for enhancing yeast strain performance in high-ethanol environments. Our findings not only contribute to the fundamental understanding of stress response mechanisms in yeast but also offer practical implications for strain engineering in the biotechnology and beverage industries. The unexpected magnitude of the Ino2p variants’ effect on ethanol tolerance underscores the importance of considering strain-specific genetic backgrounds in metabolic engineering strategies.

## INTRODUCTION

During fermentation, yeast cells are dynamically exposed to various interrelated stresses, including osmotic (1), oxidative (2), thermal (3), ethanol (4), and starvation (5). These stress conditions can significantly affect the yeast population and fermentation efficiency (6). Yeast cells have evolved to be exceptionally capable of surviving these sudden and harsh changes. The environmental stress response (ESR) in yeast includes transcriptional reprogramming of hundreds of genes (7, 8). Key transcription factors, such as *MSN2/4*, *HSF1*, and *YAP1*, coordinate this response (9). They activate various protective mechanisms, such as the accumulation of heat shock proteins, modulation of the cell cycle, membrane sterol modifications, and trehalose production, to stabilize proteins and membranes (10, 11).

Ethanol toxicity is the primary fermentation stressor, adversely affecting microbial growth, cell cycle control, and metabolic activity, with direct consequences for productivity and yields (12). Consequently, stress tolerance mechanisms are crucial for the efficiency of yeast cell growth and metabolism (12). Robust strain platforms have improved fermentation kinetics and ethanol yields (13); however, optimizing production remains a key challenge, underscoring the need for further research to enhance strain performance (14).

Recent studies have shed light on the complex mechanisms of ethanol tolerance in *Saccharomyces cerevisiae*. Transcriptomic and proteomic analyses reveal that ethanol stress induces filamentous growth and sexual reproduction and activates G-protein-coupled receptor signaling and metal ion regulation, while implicating mitochondria and endoplasmic reticulum as key responders (15). In addition, aneuploidy, particularly polysomic chromosome III, has been identified as a key factor in enhancing ethanol tolerance (10). Besides, long non-coding RNAs (lncRNAs) have emerged as important regulators in ethanol stress response, acting on various stress-responsive systems in a strain-specific manner (16, 17). These lncRNAs interact with proteins involved in cell wall maintenance, cell cycle, growth, longevity, and metabolic processes. Additionally, sphingolipid metabolism, peroxisome function, and energy metabolism have been implicated in driving ethanol tolerance phenotypes (16). Furthermore, these lncRNAs can modulate gene expression through cis-regulation of adjacent genes or trans-regulation of distant genes (18).

Another important factor linked to ethanol tolerance in various *Saccharomyces* strains is the fatty acid composition of lipid membranes (19–24). Related to this mechanism, a previous study characterized the ethanol tolerance of 61 *S*. *cerevisiae* strains, from which five strains were selected for detailed analysis of their membrane composition (22). Among these, three strains are particularly relevant to the current investigation: the highly tolerant AJ4, the slightly ethanol-tolerant MY26, and the strain MY3, which showed high tolerance in solid media but low tolerance in liquid media. Notably, AJ4 demonstrated the greatest capacity to modulate its membrane fluidity in response to ethanol exposure, exhibiting significantly increased fluidity at 10% ethanol and forming the “leakiest” liposomes when challenged with ethanol. Additionally, AJ4 had a lower content of phosphatidylethanolamine (PE) compared to the other strains. In contrast, MY26, the least tolerant strain, displayed no changes in membrane fluidity across varying ethanol concentrations, had a higher PE content, and produced less permeable liposomes. MY3 appeared to fall between AJ4 and MY26 in terms of membrane adaptability. These findings suggest that the ability to adjust membrane fluidity and lipid composition is a crucial mechanism for ethanol tolerance in yeast strains. The capacity to create more fluid membranes likely enhances tolerance to ethanol’s disruptive effects, contributing to improved resistance against ethanol stress (22).

However, the precise mechanisms underlying these associations require further elucidation, particularly concerning gene expression changes in lipid biosynthesis. While lncRNAs offer exciting new avenues for research, studying well-characterized mRNAs and their regulatory networks provides a more comprehensive understanding of ethanol tolerance mechanisms and offers immediate practical applications for strain improvement. This study aimed to delve deeper into the molecular mechanisms and pathways responsible for varying ethanol tolerances in three *S*. *cerevisiae* strains that have previously shown both different lipid composition and ethanol tolerance. We hypothesized that these tolerance mechanisms are primarily related to membrane lipid composition and the regulation of genes involved in lipid synthesis. What sets this study apart is the use of previously characterized *S. cerevisiae* strains (MY3, MY26, and AJ4) with known ethanol tolerance and membrane composition. To explore these hypotheses, we employed a combination of transcriptomic analysis and phenotypic characterization. This approach enabled us to capture dynamic changes in gene expression in response to ethanol stress. We identified two functionally critical amino acid substitutions in the transcription factor *INO2* (V263I and H86R) that distinguish the AJ4 strain. These mutations modulate ethanol tolerance by altering the expression of genes involved in ergosterol biosynthesis and other lipid metabolic pathways. Ultimately, this research aims to enhance our understanding of yeast biology and improve the efficiency of ethanol production in biotechnological applications, thereby making a vital contribution to the field.

## RESULTS

### Transcriptomic analysis of *S. cerevisiae* strains with varying ethanol tolerance during their growth in ethanol media

To determine transcriptomic differences in response to ethanol, we selected three *S*. *cerevisiae* that exhibit varying levels of ethanol tolerance: AJ4, a highly tolerant strain derived from a commercial fermentation process; MY3, a moderately tolerant strain used in the production of rosé and red wines; and MY26, a slightly tolerant strain isolated from agave fermentation in Mexico (22). The transcriptomes of the selected strains were analyzed at three time points, using the beginning of the fermentation, before ethanol addition, as a reference (*t0*). The first samples were taken at the early exponential phase (EEP = *t1*), the second at the late exponential phase (LEP = *t2*), and the last at the stationary phase (SP = *t3*). Selecting these three times for our study on ethanol tolerance allows for a comprehensive analysis of growth dynamics and stress responses. Each strain exhibited distinct behaviors under varying ethanol conditions, necessitating the selection of specific sampling times that reflect these differences. Sampling at EEP reveals initial adaptive responses, while LEP highlights mid-exponential growth dynamics, and SP assesses long-term effects on cell viability. By correlating physiological states with specific phenotypic traits, this multi-point strategy enhances data reliability and interpretation, ultimately contributing to a better understanding of ethanol tolerance mechanisms in yeast. The three strains showed similar growth when no ethanol was added. The specific growth rates and lag times, respectively, were AJ4 = 0.13 ± 0.01 h^−^¹, 4.8 ± 0.2 h; MY26 = 0.88 ± 0.02 h^−^¹, 3.07 ± 0.2; and MY3 = 0.9 ± 0.005 h^−^¹, 3.5 ± 0.01. Under ethanol stress, the three strains exhibited markedly different growth dynamics. At 6% ethanol, AJ4 and MY26 displayed comparable specific growth rates (0.11 ± 0.007 h^−^¹ and 0.09 ± 0.02 h^−^¹, respectively), while MY3 was more affected, with a reduced μ of 0.069 ± 0.03 h^−^¹. Lag phases were similar across strains at this concentration. At 10% ethanol, growth was significantly impaired in all strains. MY26 showed the highest specific growth rate (0.15 ± 0.1 h^−^¹), but this was accompanied by an exceptionally long lag phase of 35 ± 5.4 h, substantially delaying the onset of growth, and a maximum OD_600_ of 5.62 ± 0.44. AJ4, in contrast, had a lower μ (0.052 ± 0.01 h^−^¹) but a much shorter lag phase (4.4 ± 0.4 h) and a higher MaxOD (8.97 ± 1.44), enabling it to resume growth considerably earlier and at a higher rate. MY3 was the most sensitive strain, with a very low μ (0.014 ± 0.003 h^−^¹) and a moderate lag phase of 6.57 ± 2.27 h.

**Fig 1:**
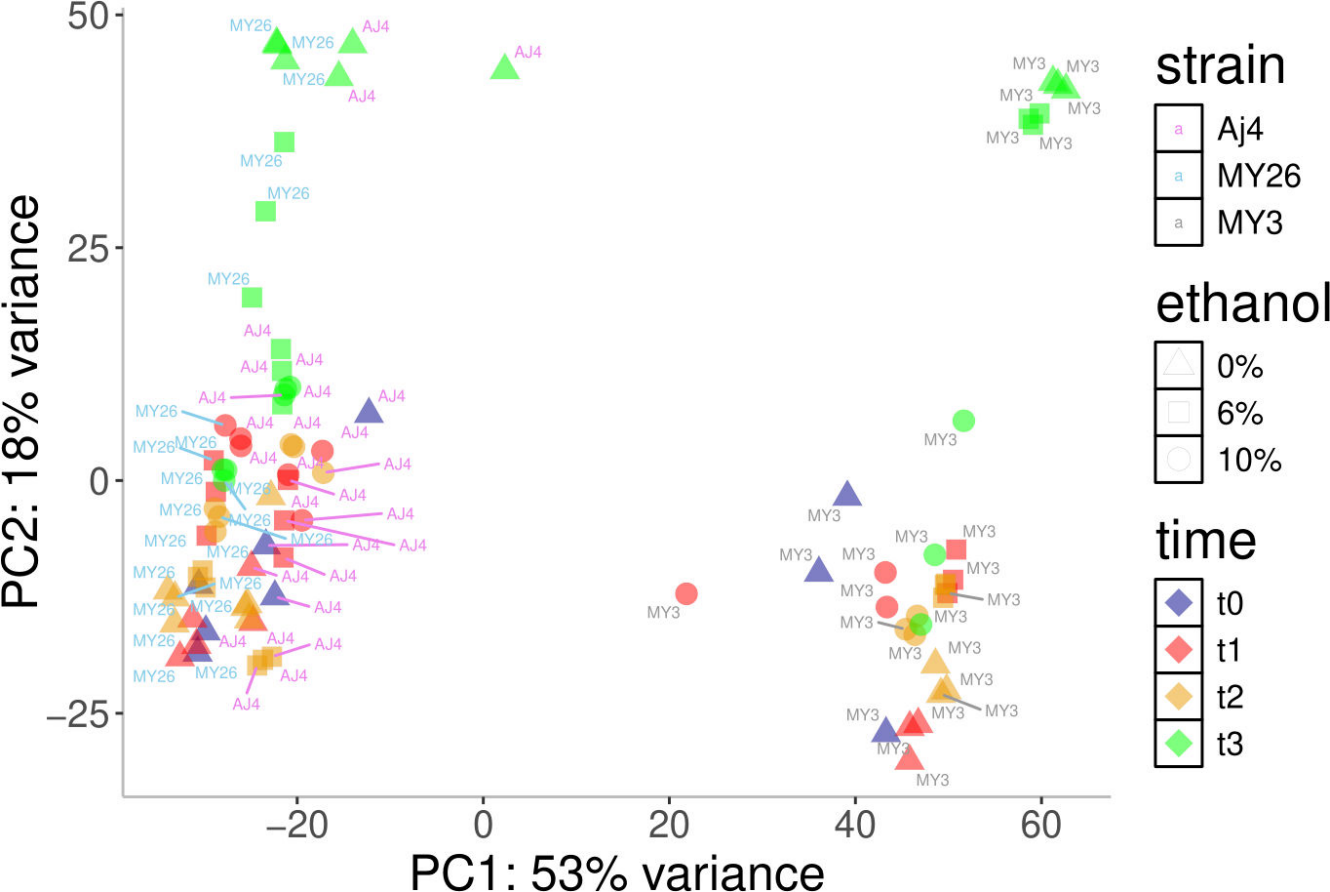
PCA based on the top 500 genes with the highest expression variability across all samples. *S. cerevisiae* strains AJ4, MY3, and MY26 are represented by circles with purple, blue, and yellow outlines, respectively. Ethanol concentrations of 0%, 6%, and 10% are indicated by blue, yellow, and red circle filling, respectively.

Transcriptomic analysis was performed, using *t0* as a control, to identify differentially expressed (DE) genes among strains at each fermentation stage and varying ethanol concentrations. Principal component analysis (PCA) of all samples showed that they primarily clustered by strains (Fig. 1). Samples at *t3* were more distinct, especially without ethanol. For *t3* samples with 6% or 10% ethanol, the separation depended on the strain. As PCA was based on the top 500 genes presenting more expression variability among all samples, it seems that the changes provoked by the variable “ethanol concentration” are masked by the ones by the variable “strain,” or even the variable “growth phase”.

To focus on the ethanol effect in each strain, differential expression at every time point was assessed with 6% and 10% ethanol compared to 0% ethanol. The number of DE genes increased over time, likely due to a decrease in the synchronization of the cells’ physiological state. This was partly a consequence of the transcriptomic response to ethanol, which began when different ethanol concentrations were added, leading to a complex gene regulation cascade.

Starting with the analysis at *t1*, it is noteworthy that the stress-responsive genes *HSP26*, *HSP32,* and *SSA3* were strongly overexpressed (log2 fold > 2.5) in all three strains under 10% ethanol stress. However, under 10% ethanol at *t1*, strains MY3 and MY26 shared another set of highly overexpressed genes related to sporulation and mating: *ADY2, GAS4, HO, MAM1, MND1, PRM1, SGA1, SHC1, SPO13, SPS1,* and *SPS100*. This suggests that less tolerant strains prepare to produce spores to endure highly stressful conditions for long periods, while AJ4 can cope without such preparation. In contrast, at 6% ethanol, the only gene commonly overexpressed among the three strains was *FMP45*. In this condition, the stress-responsive genes *DDR2* and *HSP12* were overexpressed in the two most sensitive strains, MY3 and MY26. For AJ4, the overexpressed genes included *CTR1, CTR3, FET3, FIT2*, *FRE1, HSP26, HSP30,* and *SSA4*. At 10% ethanol, *t1*, among the strongly repressed genes (log2 fold change < −2.5), the sensitive strains MY3 and MY26 shared more repressed genes than AJ4. These genes included *ADE1, ADE2, ADE13, ADE17, COB, COX1, COX2,* and *COX3*.

At *t2*, under 10% ethanol, few genes were strongly overexpressed in all three strains; however, the stress response genes *HSP12* and *DDR2* were consistently identified. Among the repressed genes at *t2* under 10% ethanol, *AGP1, DUR1,2, GAP1,* and *MEP2* were repressed in all strains. Additionally, strains MY3 and MY26 had *GAL2* and *HXT2* repressed.

At *t3*, the unspecific hexose transporters *HXT3* and *HXT4* were overexpressed in all strains. *ELO3* was also overexpressed in the three strains, while *ELO2* was exclusively overexpressed in the sensitive strains MY3 and MY26. A notable response at *t3* under 10% ethanol was the downregulation of aerobic respiration and fatty acid beta-oxidation in all strains, especially in MY3 and MY26.

A GO term enrichment analysis (https://doi.org/10.5281/zenodo.16876800) revealed commonalities among the three strains, including significant rewiring of nitrogen and carbon metabolism, as well as repression of genes involved in lipid synthesis and membrane regulation pathways, such as the tricarboxylic acid cycle and ergosterol synthesis. Stress response and protein refolding were also prominent.

Thus, we observed that the response to ethanol involves elements of the general stress response, which have varying consequences depending on the strain. Plasma membrane regulation may play a crucial role in this response.

### Importance of plasma membrane and lipid metabolism in strain-specific response to ethanol

**Fig 2:**
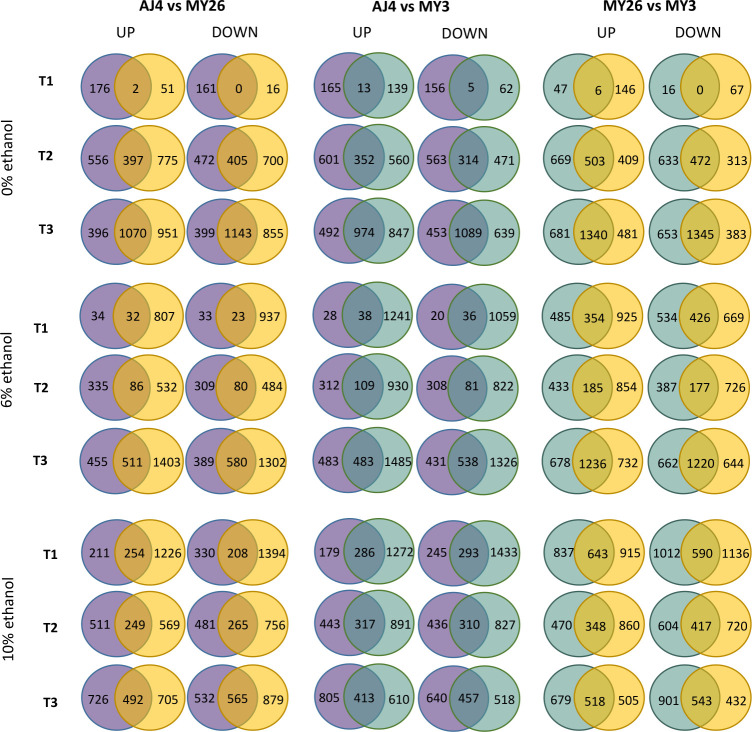
A Venn diagram illustrates the genes uniquely upregulated and downregulated in *S. cerevisiae* strains AJ4, MY3, and MY26 under three ethanol concentrations, at three time points. The strains were grown in GPY media containing 0%, 6%, and 10% ethanol, with samples collected at times *t1*, *t2*, and *t3*, and compared to *t0*. Differentially expressed genes were identified through DE analysis, applying a threshold for an adjusted *P*-value <0.05 (Benjamini-Hochberg correction).

To examine how gene expression varies at different stages of growth in comparison to a control situation for each strain, we established *t0* as the reference point in our experimental design. Specifically, we analyzed DE genes at each time point and ethanol condition by comparing each strain to the other two. This analysis involved identifying the intersection of DE gene sets. The large number of genes exclusive to each strain indicates highly variable transcriptome profiles among the studied strains (Fig. 2).

Due to the large input sample sizes for enrichment analyses and the application of Bonferroni corrections, some cellular functions with DE genes may be masked in our results. Many of the identified categories are directly related to general stress response, cell division, ribosomal translation, and central carbon metabolism. These functions are integral to stress response and growth impairment, which were anticipated under the experimental conditions. The more sensitive strains, MY3 and MY26, exhibited many of these functional categories from their exclusively DE genes in comparison to AJ4.

Interestingly, numerous other groups of GO terms were related to both plasma membrane elements and lipid biosynthesis. The “plasma membrane-enriched fraction” term was obtained from AJ4 repressed genes that were not present in MY3 or MY26. However, terms like “membrane,” “integral to membrane,” and “plasma membrane” were also found for MY26 and MY3. Additionally, the categories “cellular lipid metabolic process” or “lipid biosynthetic process” were present in all three strains. Delving into more specific metabolic pathways, the “phospholipid biosynthetic process” term was identified in MY26 upregulated genes, distinct from MY3 at *t1* with 6% ethanol. This may indicate plasma membrane adjustments. However, ergosterol biosynthesis was the most recurrent family of GO terms for MY3 and MY26 repressed genes compared to AJ4, appearing at both ethanol concentrations at different time points. While translation or cell cycle arrest can result from ethanol-induced stress, the plasma membrane acts as the first barrier against ethanol, suggesting that yeasts may adjust to ethanol toxicity through plasma membrane remodeling.

**Fig 3:**
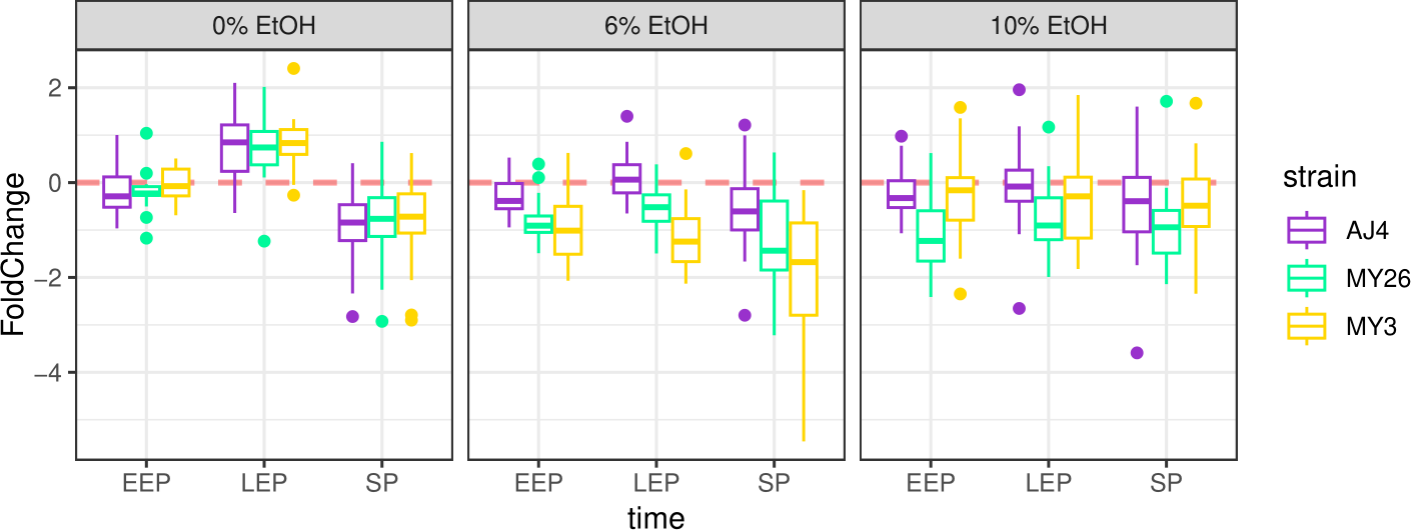
Results from the fold change analysis of ergosterol synthesis genes in *S. cerevisiae* strains AJ4, MY3, and MY26 were evaluated under three ethanol concentrations (0%, 6%, and 10%), and at three time points (*t1, t2, t3*), relative to the baseline expression level at *t0*, without ethanol supplementation. Paired *t*-tests revealed significant differences between ethanol-tolerant strain AJ4 and less-tolerant strains MY26 and MY3. Significant adjusted *P*-values: AJ4-MY3 at 6% LEP = 0.0000124; AJ4-MY26 at 10% EEP = 0.003; AJ4-MY3 at 6% SP = 0.006; AJ4-MY3 at 6% EEP = 0.013; AJ4-MY26 at 6% LEP = 0.019; AJ4-MY26 at 6% EEP = 0.039.

Consequently, genes related to lipid metabolism and membrane homeostasis were investigated in detail by filtering out all the genes not included in the functional categories “plasma membrane” (GO: 0005886) or “lipid metabolism” (GO: 0044255) from the Gene Ontology. Interestingly, under 6% and 10% ethanol conditions, many genes coding for enzymes involved in ergosterol biosynthesis were repressed at every time point in the sensitive strains MY26 and MY3, but not in AJ4. Notably, some conditions, such as 6% ethanol in LEP, showed significant differences, while other cases exhibited non-significant yet divergent trends with greater variability. In contrast, the expression of these genes was similar for all strains in the absence of ethanol (Fig. 3). Examining the expression of each gene in the pathway revealed that most of them were repressed in MY26 and MY3 under ethanol conditions, rather than just one or a few genes standing out.

**Fig 4:**
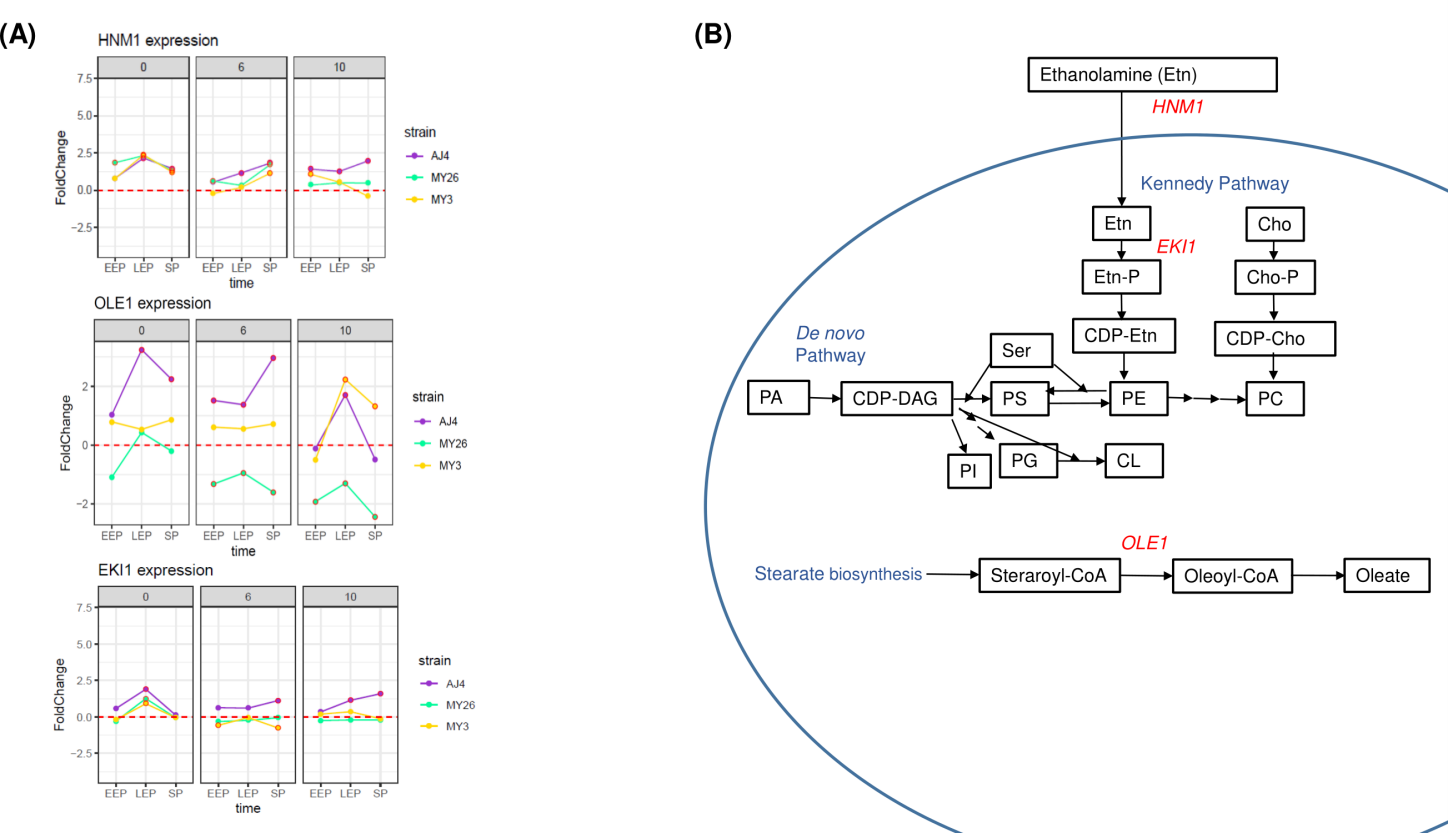
(**A**) Results of the expression analysis of genes *HNM1*, *EKI1*, and *OLE1* in *S. cerevisiae* strains AJ4, MY3, and MY26 were obtained under three ethanol concentrations (0%, 6%, and 10%), and at three time points (*t1*, *t2*, *t3*), relative to the baseline expression level at *t0*, without ethanol supplementation. (**B**) A schematic representation of the phospholipid synthesis pathway involving the enzymes encoded by genes *OLE1*, *HNM1,* and *EKI1*. (Etn-P, phosphoethanolamine; CDP-Etn, CDP-ethanolamine; Cho, choline; Cho-P, phosphocoline; CDP-Cho, CDP-choline; PC, phosphatidylcholine; PS, phosphatidylserine; Ser, serine; CL, cardiolipin; PG, phosphatidylglycerol; PI, phosphatidylinositol; CDP-DAG, CDP-diacylglycerol; PA, phosphatidic acid).

Regarding the main membrane lipid biosynthesis processes, other genes exhibited differential behavior among strains. This includes *HNM1*, a transporter of phospholipid precursors such as choline and ethanolamine, and *EKI1*, the first enzyme in the transformation of ethanolamine into phosphatidylethanolamine. Both genes exhibited similar expression dynamics in the absence of ethanol for all three strains; however, their expression changed in the presence of ethanol, with AJ4 maintaining higher transcriptional levels for both genes (Fig. 4). Additionally, *OLE1*, responsible for the desaturation step in the synthesis of oleic and palmitoleic acids, was repressed in MY26 under alcoholic conditions. In contrast, AJ4 and MY3 present higher expression levels (Fig. 4).

### The role of the transcription factor Ino2p in the regulation of the response to ethanol stress

**Fig 5:**
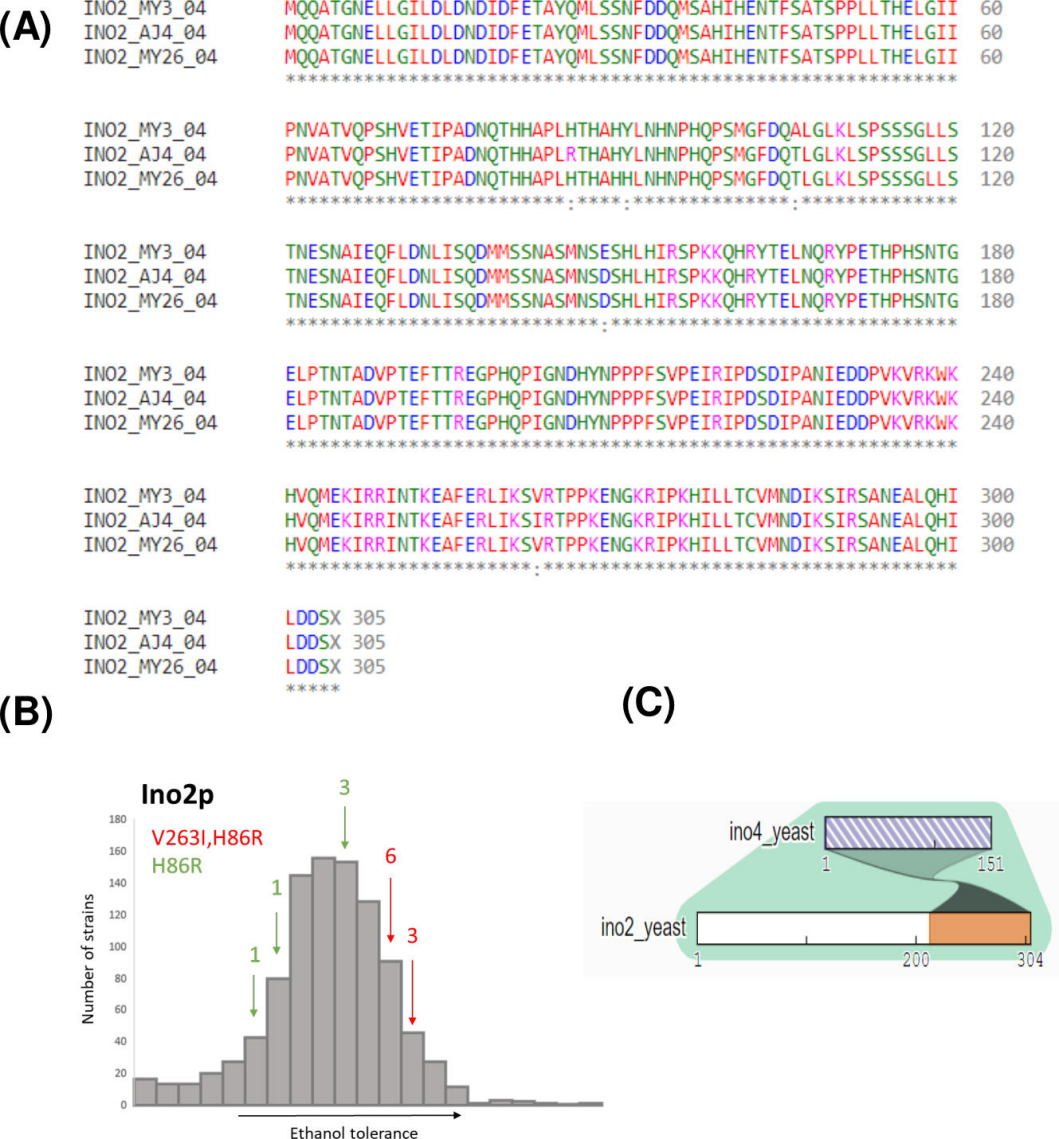
(**A**) Alignment of the amino acid sequence of Ino2p transcription factor from *S. cerevisiae* strains AJ4, MY3, and MY26. (**B**) Representation of the ethanol tolerance of 979 strains (27), indicating the presence of mutations in V263I and in H86R in Ino2p. (**C**) Representation of the interaction between Ino2p and Ino4p.

Remarkably, a transcriptional factor enrichment analysis made with Yeastract, using “DNA binding and expression evidence” (25), indicated that our set of differentially expressed genes is regulated by the transcription factor encoded by the gene *INO2*. A deeper investigation into *INO2* (*YDR123*) involved a comparative analysis of the Ino2p protein sequence for the selected strains. The result revealed two variants specific to AJ4 compared to the other two strains: a histidine-to-arginine substitution at position 86 (H86R) and a valine-to-isoleucine substitution at position 263 (V263I). Notably, Clustal Omega (26), a multiple sequence alignment tool, classified these amino acid replacements as conservative, indicating that they are likely to preserve the protein’s overall structure and function. Furthermore, we analyzed 979 Ino2p sequences from different strains, a highly representative set of *S. cerevisiae*’s diversity, from 1,011 strains from Peter et al. (27) , to investigate the prevalence of the mutations observed in AJ4 across other strains. The V263I variant was identified in only nine strains, while the H86R variant was found in the same nine plus five additional strains. To explore the potential impact of these variants on ethanol tolerance, we referred to phenotypic data from Peter et al. (27), which included growth assays in media containing 15% ethanol. Strains exclusively harboring the H86R mutation exhibited relatively moderate ethanol tolerance. In contrast, strains carrying both mutations demonstrated noticeably higher ethanol tolerance levels, surpassing the median tolerance threshold (Fig. 5). Thus, the AJ4 *INO2* allele could be a significant contributor to the strain’s higher ethanol tolerance.

**Fig 6:**
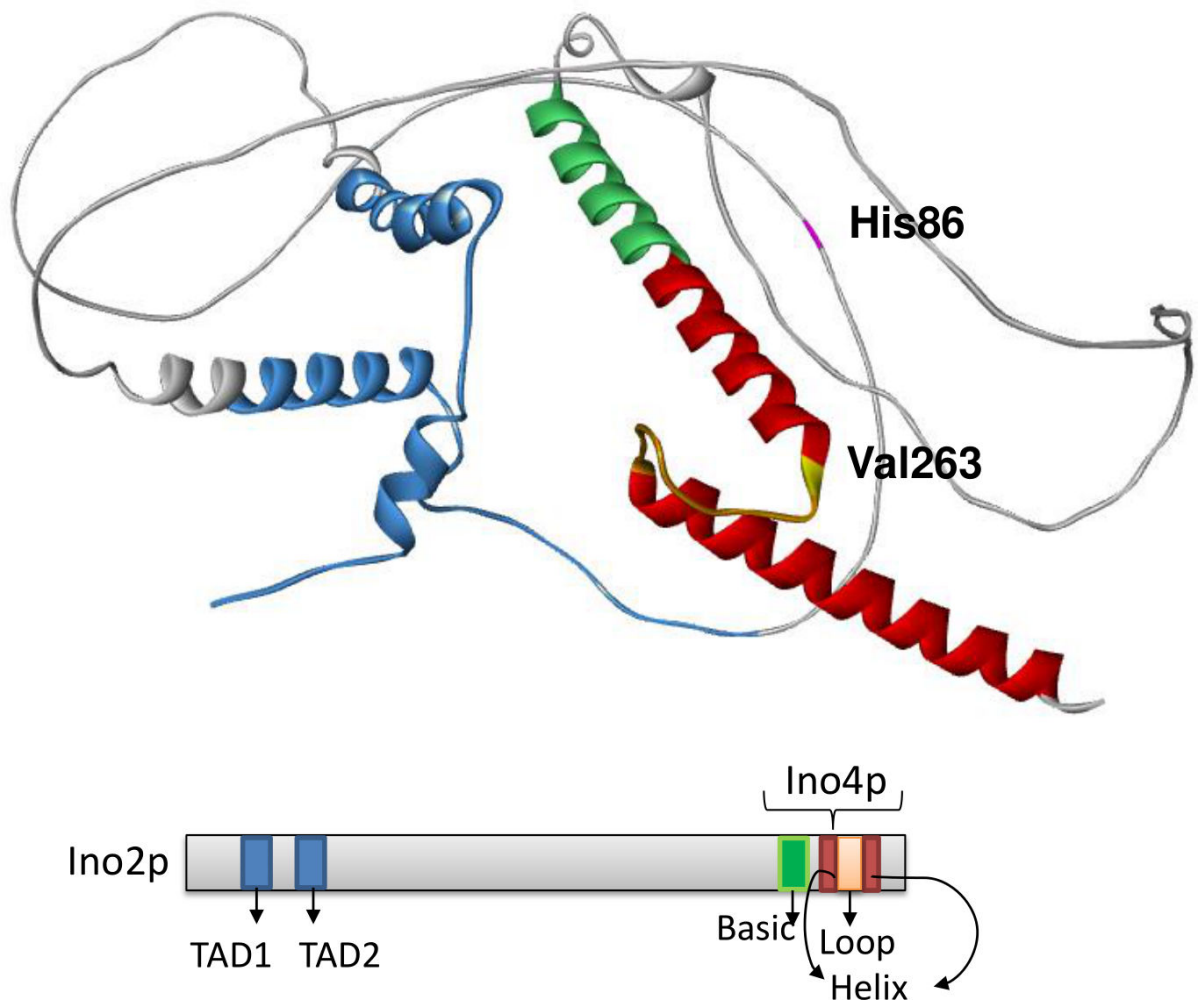
Structure of the transcription factor Ino2p. This structure was predicted using AlphaFold (28) and visualized with BIOVIA Discovery Studio (29). The TAD1 and TAD2 regions are depicted in blue; the basic domain where the bHLH begins is shown in green, and the helix of the domain is highlighted in red. Amino acids 86 and 263 are marked in pink and yellow, respectively.

The Ino2p^R86H^ variant features the amino acid replacement in a long loop within the N-terminal region of the protein. The substitution of histidine with arginine in a protein loop can have various effects depending on the specific location and the role of the residue in the protein’s structure and function. Differences in charge, size, flexibility, and hydrogen bonding potential between histidine and arginine may result in changes to protein stability, folding, and interactions. On the other hand, the AJ4 Ino2p^I263V^ variant has its amino acid replacement located at the beginning of the helix that interacts with Ino4p, near the ligand-binding site, as observed in the predicted structure (Fig. 6). Although the replacement of valine with isoleucine is considered conservative, it can still affect protein structure, stability, and function, depending on the specific location and context of the amino acid replacement. The additional methylene group in isoleucine may subtly alter the packing and hydrophobicity of the side chain, potentially leading to functional consequences. To investigate whether the Ino2p variants H86R and V263I impact *S. cerevisiae*’s ethanol tolerance, we conducted experimental tests.

### Ethanol tolerance in *INO2* mutants

**Fig 7:**
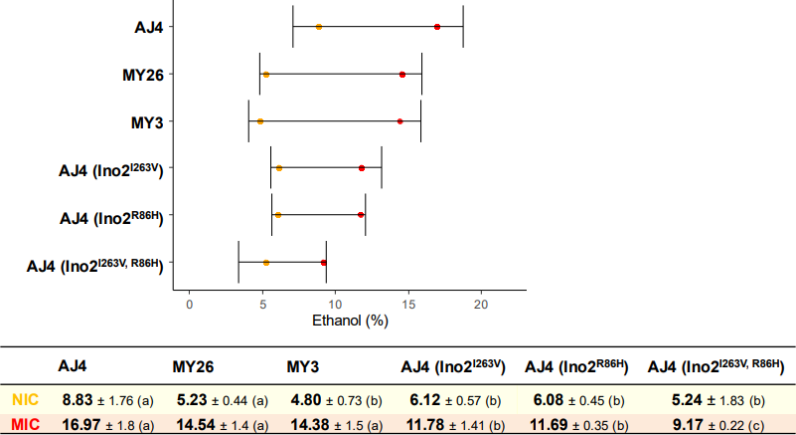
Ethanol NIC and MICs of wild-type AJ4, and the mutants AJ4 (Ino2p^I263V^), AJ4 (Ino2p^R86H^), and AJ4 (Ino2p^I263V, R86H^).

To investigate whether V263I and H86R replacements could affect ethanol tolerance, we generated *INO2* mutants using CRISPR-Cas9 in the tolerant strain AJ4. The three mutants generated were AJ4 (Ino2p^I263V^), AJ4 (Ino2p^R86H^), and AJ4 (Ino2^I263V, R86H^). Ethanol tolerance tests were then conducted at various concentrations to determine growth parameters. We calculated the areas under the growth curve (AUC) at different ethanol concentrations and used these to determine the non-inhibitory concentrations (NICs) and maximum inhibitory concentrations (MICs) (Fig. 7).

All mutants exhibited decreased NIC and MIC values, clearly indicating that both amino acid replacements contribute to ethanol tolerance in AJ4. The double mutant showed a more pronounced effect, with NIC values similar to those of the moderately and slightly tolerant strains, MY3 and MY26, and MIC values significantly lower. These findings suggest that the presence of this *INO2* allele, encoding two amino acid replacements, could explain not only the differences in ethanol tolerance between AJ4 and the other two strains but also a more severe effect in the AJ4 genomic background. This modification likely impaired crucial functions, such as lipid biosynthesis regulation and transcriptional control of genes involved in ethanol tolerance.

In summary, the reversion of the AJ4 V263I and H86R replacements was observed to decrease ethanol tolerance, with a more pronounced effect at higher ethanol concentrations. This reduced tolerance may result from changes in the interaction between Ino2p and other transcription factors, such as Ino4p and Toa1p, which are involved in regulating genes related to membrane lipid biosynthesis.

### Expression of *ELO1, HNM1*, *EKI1*, and ergosterol genes in *INO2* mutants

**Fig 8:**
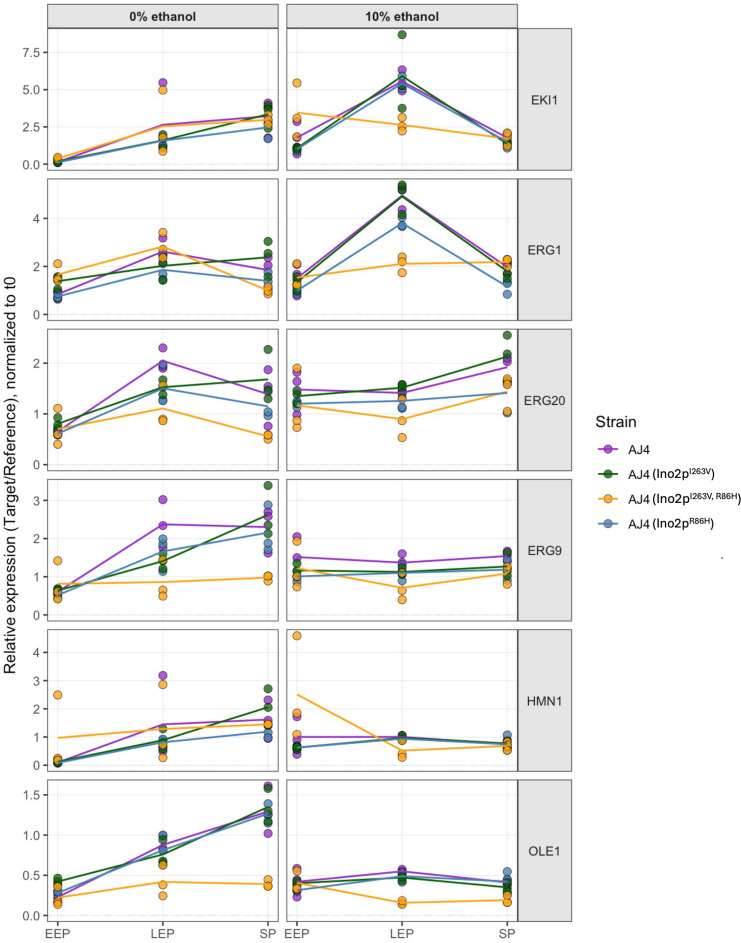
Differential expression of ergosterol and lipid metabolism genes (*ERG1*, *ERG9*, *ERG20*, *EKI1*, *HNM1*, and *OLE1*) in AJ4 (purple), *INO2* mutants AJ4 (Ino2p^I263V^) (green), AJ4 (Ino2p^R86H^) (blue), and AJ4 (Ino2p^I263V, R86H^) (orange) under ethanol stress (10% vol/vol) and control conditions across growth phases. The target:reference expression ratio was normalized to *t0*, with statistical data available in https://doi.org/10.5281/zenodo.16876800. Individual replicates are depicted by points colored according to the respective strain, with the corresponding media indicated.

Given the lower ethanol tolerance observed in Ino2p mutants, we sought to investigate how the V263I and H86R changes in AJ4’s Ino2p affect its regulatory function on genes differentially expressed in AJ4, MY3, and MY26 strains, which have varying ethanol tolerance and lipid membrane compositions. To test this hypothesis, we cultured AJ4 and its mutant derivatives AJ4 (Ino2p^I263V^), AJ4 (Ino2p^R86H^), and AJ4 (Ino2^I263V, R86H^), with 10% of ethanol and without ethanol, and we measured the expression of *ELO1*, *HNM1*, *EKI1*, and ergosterol biosynthesis genes (*ERG1*, *ERG9*, and *ERG20*). Gene expression analysis by qPCR (target:reference gene ratios) was normalized to *t0* values for each growth stage (https://doi.org/10.5281/zenodo.16876954). The results demonstrated that ethanol exposure triggered dynamic, strain-specific transcriptional responses (Fig. 8). For the transcription factor *EKI1,* all strains showed stage-dependent upregulation in the absence of ethanol, with AJ4 (Ino2^I263V,R86H^) showing significantly higher expression levels during EEP compared to the other strains. When ethanol was added, AJ4 (Ino2^I263V,R86H^) displayed a unique *EKI1* expression pattern, characterized by an early transient peak during LEP that was significantly lower than in other strains. In contrast, all other strains reached their maximal *EKI1* expression levels at LEP before subsequent downregulation. The *ERG* gene family (*ERG1*, *ERG9*, *ERG20*), involved in sterol biosynthesis, displayed different expression profiles. In the case of *ERG1*, when ethanol was added, all strains showed increased expression during LEP, except for the AJ4 revertant mutant AJ4 (Ino2^I263V,R86H^). This mutant displayed significantly lower *ERG1* expression at LEP, showing only minimal change before increasing during SP. Both *ERG9* and *ERG20* presented significant differences at LEP and SP between AJ4 and (Ino2^I263V,R86H^) under ethanol-free conditions. However, when ethanol was added, both genes exhibited similar expression patterns across strains, though with substantial inter-replicate variability. *HNM1* expression showed no significant differences between strains under media without ethanol addition. Nevertheless, ethanol stress revealed lower expression in AJ4 (Ino2^I263V, R86H^) at LEP. In the case of *OLE1*, all strains exhibited gradual upregulation in the absence of ethanol, except for AJ4 (Ino2I263V, R86H), which showed significantly lower expression at LEP and SP. Ethanol exposure reduced *OLE1* expression, with AJ4 (Ino2^I263V, R86H^) showing the lowest expression.

To summarize, while no universal expression pattern was observed across strains or genes, the Ino2^I263V,R86H^ mutation in AJ4 demonstrably altered the expression of key metabolic regulators, including *ERG1*, *ERG9*, *and ERG20,* which play critical roles in ergosterol biosynthesis, as well as *EKI1, HNM1,* and *OLE1,* affecting lipid metabolism.

## DISCUSSION

Ethanol stress represents a significant challenge for yeast cells in industrial biotechnology, underscoring the importance of elucidating the mechanisms involved in ethanol stress response (17). Elucidating these mechanisms is vital for developing targeted strategies to enhance yeast strains and optimize processes for improved sustainability (30). In this study, we comprehensively analyzed gene expressions under different ethanol concentration stress in three *S*. *cerevisiae* strains: the highly tolerant AJ4, the moderately tolerant MY3, and the slightly tolerant MY26. We observed some general stress responses, including mechanisms to avoid protein misfolding and aggregation and to maintain cellular homeostasis (31). Additionally, we found that genes related to sporulation and mating were overexpressed, suggesting that yeast cells might enter a quiescence state, waiting for better conditions for vegetative growth, as reported in other studies on stress response (32). We also identified genes related to mitochondrial aerobic respiration, which is essential for energy generation (31) and maintaining cellular homeostasis under ethanol stress. Furthermore, sugar transporters, including *GAL2, HXT2, HXT3,* and *HXT4* (33), showed differential expression and have been previously linked to ethanol tolerance (34).

Our main focus, however, was on the effect of ethanol stress on yeast membrane lipid metabolism genes, as changes in the membrane lipid composition are the first barrier against ethanol, and the strains used in this study were selected due to their differences in lipid membrane composition. We revealed that genes involved in inositol monophosphate synthesis were overexpressed in the tolerant strain AJ4, suggesting that maintaining lipid homeostasis is crucial in ethanol tolerance response (22). *ELO3*, which encodes a fatty acid elongase for synthesizing very long-chain fatty acids (20–26 carbons) from C18-CoA primers, was overexpressed in all three strains at the time point *t3*. Additionally, *ELO2,* encoding an elongase for fatty acids up to 26 carbons, was overexpressed in MY3 and MY26. Long-chain fatty acids are known to reduce membrane fluidity (35), a trait commonly associated with increased ethanol tolerance (23). Furthermore, sphingolipids, especially long-chain fatty acids like C26-phytosphingolipid species, interact with ergosterol to form lipid rafts critical for membrane organization. Ergosterol synthesis was the primary metabolic pathway repressed in MY3 and MY26 compared to AJ4 under ethanol stress. Ergosterol is the main sterol found in yeast cells, playing a critical role in regulating yeast membrane permeability and fluidity, particularly under high ethanol stress (36). By incorporating ergosterol into the lipid bilayer, it influences the packing and ordering of membrane lipids, thereby affecting the membrane’s physical properties. Traditionally, reducing membrane fluidity and permeability has been considered a key strategy for enhancing ethanol tolerance (37–39). In our study, the strain-specific upregulation of ergosterol biosynthesis genes in the highly tolerant strain AJ4 aligns with its role in enhancing membrane compactness to achieve higher ethanol tolerance. However, previous research on *in vitro* membranes derived from AJ4 lipid extracts suggested that increased membrane fluidity contributes to a higher ethanol tolerance in AJ4 (22). This apparent contradiction may be due to the complexity of plasma membrane organization *in vivo*, where different subregions (rafts or islands) have variable compositions and, consequently, distinct physical properties (40). One such ergosterol-rich subregion is associated with Can1p, a membrane arginine permease that requires phosphatidylcholine for its localization and is, therefore, exclusively found in lipid rafts. This specific association has been linked to increased tolerance to ethanol and other stresses (38). A higher ergosterol concentration in these subregions could be compatible with an overall increase in membrane fluidity elsewhere. Phospholipids, particularly PE, are associated with ethanol tolerance, with higher PE concentrations found in less tolerant strains (22). The ratio of sterols to phospholipids significantly influences membrane fluidity and ethanol tolerance (41). This is reflected in our study, where the differential expression of genes involved in membrane lipid biosynthesis is observed among strains with varying ethanol tolerance. For instance, *HNM1*, a transporter of phospholipid precursors, and *EKI1*, the first enzyme in PE synthesis, showed higher transcriptional levels in the ethanol-tolerant strain AJ4 in the presence of ethanol. Additionally, *OLE1*, responsible for the desaturation of oleic and palmitoleic acids, was repressed in the less tolerant MY26 strain under alcoholic conditions, while AJ4 and MY3 maintained higher expression levels. These findings align with the observation that increased levels of unsaturated fatty acids enhance plasma membrane fluidity, contributing to ethanol tolerance (42). Our findings demonstrated that *INO2* regulated the majority of lipid-related genes differentially expressed among strains with varying ethanol tolerance and lipid composition. The transcription factor Ino2p plays a central role in regulating phospholipid biosynthesis genes in the yeast *S. cerevisiae* (43). The lipid biosynthesis genes share a common promoter region sequence (5′CATGTGAAAT3′), recognized as the inositol-sensitive upstream activation sequence (UAS)_INO_ (44, 45). The Ino2p-Ino4p complex binds to this promoter region within the *INO1* gene, which contains the consensus-binding site for the basic helix-loop-helix (bHLH) family of transcription factors (46). This bHLH-binding site is essential for the repression and activation of *INO1* and other phospholipid biosynthesis genes. *INO1* regulation is particularly important under ethanol stress, as upregulation of lipid metabolism-related genes like *INO1* is concurrent with the activation of the unfolded protein response (13, 47). Moreover, *INO1* is regulated by the transcription factor IIA(TFIIA) subunit Toa1p, which interacts physically with Ino2p and is necessary for the proper activation of genes containing the (UAS)_INO_ promoter element, especially *INO1*. Specifically, Ino2p binds to two separate structural domains of Toa1p, the N-terminal four-helix bundle structure, required for dimerization with Toa2p, and its C-terminal β-barrel domain, contacting TBP and sequences of the TATA element (47).

Interestingly, our sequence analysis of the Ino2p in the three strains revealed that the highly ethanol-tolerant AJ4 strain harbors two amino acid replacements (V263I and H86R) compared to the less tolerant strains MY3 and MY26. We also identified these replacements in Ino2p sequences from strains with above-average ethanol tolerance (27). The V263I replacement impacts a helix in the bHLH involved in Ino2p-Ino4p interaction, potentially altering protein structure and function. Conversely, the H86R replacement is located in a loop domain of the N-terminal region and may affect binding with the TFIIA subunit Toa1p. Our study demonstrated that the reversion of these replacements in AJ4 led to a significant decrease in MIC for ethanol, suggesting that the Ino2p variant present in AJ4 plays a role in regulating genes involved in the ethanol tolerance response. Moreover, the AJ4-derived mutant with the double amino acid reversion exhibited lower ethanol tolerance and even slower fermentation growth than the less tolerant strains MY3 and MY26, indicating that the specific genetic background of each yeast strain can modulate, through other mechanisms, the effects of these genetic changes on the ethanol tolerance phenotype. Additionally, we demonstrated by qPCR that Ino2p mutations (I263V, R86H) in AJ4 altered the expression of ergosterol (*ERG1/9/20*) and lipid metabolism genes (*EKI1/HNM1/OLE1*), thereby disrupting ethanol stress responses. The double mutant exhibited delayed growth, transient *EKI1* induction, and suppressed *HNM1/OLE1* expression, indicating impaired membrane adaptation. These changes explain AJ4’s reduced ethanol tolerance compared to single mutants.

Looking at the GO terms that are different, MY3 and MY26 exhibited distinct cellular processes compared to AJ4, which contributed to their ethanol tolerance. MY26 showed upregulation of stress response and protein refolding mechanisms, critical for combating ethanol-induced protein misfolding and maintaining cellular integrity. MY3 demonstrated enrichment in mitochondrial translation and ribosome biogenesis, essential for energy production and protein synthesis under ethanol stress, as well as cell cycle regulation genes that could aid in adapting to ethanol-induced growth inhibition. These strain-specific adaptations in MY3 and MY26, which are less dependent on Ino2p modifications, allowed them to maintain higher ethanol tolerance compared to the AJ4 mutant with reverted Ino2p.

Taken all together, we identified an allele of *INO2* that confers ethanol tolerance, encoding a variant of the transcription factor Ino2p that affects the regulation of lipid-related genes. This discovery sheds light on the genetic regulation of lipid metabolism and membrane composition, offering potential advancements in biotechnological applications. Ethanol tolerance is a complex trait(48) involving multiple mechanisms where lncRNAs act in a strain-specific manner in the ethanol stress response (18). This suggests that transcriptional regulators, such as Ino2p, may modulate the expression and function of ethanol-responsive lncRNAs. The intricate interplay between trans-acting factors, such as Ino2p, and the unique genomic background of each strain likely contributes to the complex balance of ethanol tolerance mechanisms.

In addition, we observed that the number of ESR genes (7) found in our study was within the expected range based on their genome proportion. While ESR genes were not overrepresented in the overall response, several of the most highly expressed genes in the ethanol stress response were ESR genes, suggesting a significant role for these genes in ethanol tolerance. When examining strain-specific gene expression, the proportion of exclusive genes decreased in most cases, particularly those with statistically significant differences. However, exceptions were found, such as in the AJ4 strain at 6% ethanol at the LEP where AJ4 uniquely expressed certain genes not seen in other strains. The results of this study underscore the need to consider the entire genomic background when studying complex traits, such as ethanol tolerance. The evolutionary history and genetic variations in each strain create a finely tuned regulatory network that influences how specific mutations manifest, offering valuable insights for strain improvement and industrial applications.

## MATERIALS AND METHODS

### Media and strains

The diploid *Saccharomyces* strains AJ4, MY26, and MY3 have been used in this study due to their varying ethanol tolerances. In addition, their lipid compositions have been characterized by Lairón-Peris et al. (22). Strain AJ4 (Lallemand) is used for bioethanol production and exhibits high ethanol tolerance; MY3 (Lallemand) is a wine yeast used in the production of rosé and red wines and has low tolerance to ethanol; MY2, a strain used in agave fermentation, is characterized by low to medium ethanol resistance. These strains were taken from 15% glycerol stocks and were maintained in GPY media.

### Yeast culture and sampling

Strains were inoculated in 500 mL GPY and grown overnight. From the preculture, 750 mL of GPY media was inoculated to an OD_600_ of 0.2 in 1 L Erlenmeyer flasks. This was done in triplicate for each strain (AJ4, MY3, and MY26) and each condition (0%, 6%, and 10% of ethanol). The inoculated yeast cells were grown for 1 h without stress to allow adjustment to the medium. Subsequently, ethanol was added up to reach the specified concentrations. Flasks were placed in an incubator at 28°C, with orbital agitation at 150 rpm. Approximately 10^8^ cells were collected at different time points: before the ethanol addition (*t0*), early exponential phase (*t1*), late exponential phase (*t2*), and stationary phase (*t3*). Cells were harvested by centrifugation, frozen in liquid nitrogen, and stored at −80°C.

### RNA-seq analysis and data availability

RNA isolation was performed following the phenol-chloroform method (48). Cells were washed with diethylpryrocarbonate (DEPC) water and then treated with phenol tris, phenol-chloroform (5:1), and chloroform-isoamyl alcohol (24:1). The aqueous phase was transferred to clean tubes, and nucleic acids were precipitated first with LiCl and then with ethanol and sodium acetate. RNA was resuspended in RNase-free water. RNA concentration was measured using a Nanodrop, and RNA integrity was assessed with an Agilent Bioanalyzer. RNA-seq libraries were prepared with the Illumina Truseq Stranded mRNA kit and sequenced on an Illumina Hiseq 2000, obtaining 75 bp paired-end reads. Reads were quality-trimmed using Sickle (length 50, quality 23), aligned to the *S. cerevisiae* S288C reference genome using Bowtie2, and mapped reads were counted by Htseq-count (union mode) (49). Count data were imported, processed, and normalized by removing low-expressed genes and using the variance-stabilizing transformation method in DESeq2 for sample clustering (50). Differential expression analysis was conducted with the Limma package (v.3.32.2) (51). Counts were transformed to log cpm, and expression was adjusted by Limma-voom. GO term enrichment was performed with FunSpec (52), including differentially expressed genes with an adjusted *P* value <0.05 (53).

### Ino2p structure analysis

AlphaFold (28) was used to predict Ino2p structure, PDBe (54) was used for structural data, and BIOVIA Discovery Studio Visualizer (29) for structure and amino acids representation.

### Introducing point mutations in *INO2* by CRISPR-Cas9 system

The CRISPR-Cas System (55) was used in two steps for easier selection. First, we introduced kanMX in the regions surrounding codons for amino acids H86 or V263. KanMX was amplified from the plasmid pUG6A with designed primers with the *INO2* sequences using PCR with Ex Taq Polymerase (Takara). We used the plasmid pRCC_N-Ino2, which contained the gRNA targeting *INO2* point mutations and other necessary components, including natMX6. This plasmid was created by amplifying pRCC_N using PCR with Phusion polymerase (Thermo Fisher Scientific). Primers contained the gRNA sequence designed with Geneious software. Then, strain AJ4 was transformed using the LiAc/SS Carrier DNA/PEG method (56), introducing the plasmid pRCC_N-Ino2 and the kanMX DNA fragment. Cells were plated in GPY with 300 ng/µL nourseothricin (ClonNAT). After 2 days, colonies were replated on media with 300 ng/µL G418 to select kanMx-resistant colonies. These colonies were tested by PCR with custom-designed primers to confirm kanMX replacement of *INO2*. Selected colonies were cleaned of the plasmid with ClonNAT resistance. In step 2, we introduced the point mutation with CRISPR-Cas9 in the *INO2*-KanMX mutants. The fragment of DNA was designed by combining single sequences (forward and reverse) containing the desired mutation in the middle. The plasmid pRCC_N- kanMX-gRNA, containing the gRNA targeting kanMX, was used for transformation. Cells were plated on ClonNAT, and mutants were confirmed by PCRs with selected primers. The positive mutants were preserved in 15% glycerol at −80°C. Single mutants were used to create double mutants using the same procedure. Final mutants were AJ4 (Ino2p^I263V^), AJ4 (Ino2p^R86H^), and AJ4 (Ino2p^I263V, R86H^).

### Ethanol tolerance assay

Mutants AJ4 (Ino2p^I263V^), AJ4 (Ino2p^R86H^), and AJ4 (Ino2p^I263V, R86H^) and wild-type strains AJ4, MY26, and MY3 were cultured in 1 mL of GPY. After 16 h, cultures were centrifuged, washed, and suspended in phosphate buffered saline (PBS). After 2 h, a cell density was adjusted to 4 × 10^7^ cells/mL. Cells were inoculated into minimal medium yeast nitrogen base (YNB) with different ethanol concentrations (0, 1, 3, 5, 7, 9, 11, 13, 15, and 17%) in 96-well plates containing 220 µL of medium and 100 µL petrolatum oil (PharmpurR) to prevent ethanol evaporation.

A Stacker Microplate Handling System, attached to plate readers SPECTROstar Omega (BMG LABTECH), in a 25°C and 70% humidity chamber, measured OD_600_ to monitor growth. Microplates were shaken in orbital mode for 20 seconds each hour at 400 rpm. Yeast growth was analyzed using the Growth Curve Analysis Tool (57), and the AUC was calculated. To assess the relative growth of yeast strains at different ethanol concentrations, we computed the fractional area (fa) by comparing the AUC at each ethanol concentration to that of the control condition.


$$fa=\frac{AUC}{ControlAUC}$$

A modified Gompertz function was used to relate the fractional area (*y*) to the log of ethanol concentration (*x*) (58):


$${y=A+Ce}^{-e^{B\left(x-M\right)}}$$

In this formula, *A* is the lower asymptote of *y*, *B* is the slope parameter, *C* is the distance between the upper and lower asymptote, and *M* is the log concentration of the inflection point. The values of the NIC and MIC are described as the intersection of the lines *y* = *A* + *C* and *y* = *A*, with the equation of the line tangential to the point *M* respectively.


$$\text{MIC }{=10}^{\left(M+\frac{1}{B}\right)}\text{ NIC }{=10}^{\left(M-\frac{1\text{.718}}{B}\right)}$$

Eventually, the values of *A*, *C*, *B*, and *M* can be calculated using a non-linear fitting procedure, and NIC and MIC were determined.

### qPCR analysis of the selected genes regulated by *INO2*, in AJ4, and the reverted amino acid strains

To assess the impact of specific amino acid alterations in *INO2* on the expression of genes previously identified as differentially regulated by this transcription factor in strains with varying ethanol tolerance, we performed growth experiments under different ethanol concentrations and measured the expression levels of selected target genes.

Firstly, mutants AJ4 (Ino2p^I263V^), AJ4 (Ino2p^R86H^), and AJ4 (Ino2p^I263V, R86H^) and wild-type strains AJ4 were precultured in GPY. Then, as in the RNA-seq experiment explained above, 750 mL of GPY media was inoculated to an OD_600_ of 0.2 in 1 L Erlenmeyer flasks with 0, 6, and 10% ethanol. The conditions were exactly the same using an incubator at 28°C, with orbital agitation at 150 rpm. Samples were taken at the same time points. Then the frozen cells were subjected to RNA extraction as previously described. To convert RNA to cDNA, 0.8 mM of the four Deoxynucleoside triphosphates (dNTPs), 80 pmol Oligo(dt) was used in 2 µL of the RNA. Agarose gel electrophoresis was done to check nucleic acid contaminations and degradations. Later, cDNA was mixed with 10 mM dithiotreitol, 50 U RNAse inhibitor (Invitrogen), 1× First Strand Buffer (Invitrogen), and water up to 20 µL. We added 500U Superscript III (Invitrogen), and samples were incubated at 42 degrees for 50 min and the reaction was inactivated later. qRT-PCR was performed using the protocol used previously (59) with LightCycler Fast Start DNA Master PLUS SYBR green (Roche Applied Science, Germany) in a LightCycler 480 (Roche Applied Science, Germany) device. Oligos (https://doi.org/10.5281/zenodo.16876954) were designed to amplify *ERG1*, *ERG9*, *ERG20*, *EKI1*, *HNM1*, and *OLE1. ACT1* (actin gene), and *RDN18-1* (18S ribosomal RNA) were used as constitutive genes to normalize the amount of mRNA. Samples were processed for melting curve analysis, amplification efficiency, and cDNA concentration determination.

## Data Availability

Raw sequence data are available under BioProject ID PRJNA1150416, and the analysis is available at https://doi.org/10.5281/zenodo.16876800.
